# Two Decades of Optogenetic Tools: A Retrospective and a Look Ahead

**DOI:** 10.1002/ggn2.202500021

**Published:** 2025-09-02

**Authors:** Xiao Duan, Mo Zhu, Shiqiang Gao

**Affiliations:** ^1^ Department of Neurophysiology Institute for Physiology University of Würzburg 97070 Würzburg Germany; ^2^ College of Life Sciences Henan Normal University Xinxiang Henan 453007 P. R. China

**Keywords:** channelrhodopsin, light‐controlled protein interactions, optogenetics, photoactivated cyclases, photoreceptors

## Abstract

Over the past two decades, optogenetics has evolved from a conceptual framework into a powerful and versatile technology for controlling cellular processes with light. Rooted in the discovery and characterization of natural photoreceptors, the field has advanced through the development of genetically encoded, light‐sensitive proteins that enable precise spatiotemporal control of ion flux, intracellular signaling, gene expression, and protein interactions. This review traces key milestones in the emergence of optogenetics and highlights the development of major optogenetic tools. From the perspective of genetic tool innovation, the focus is on how these tools have been engineered and optimized for novel or enhanced functions, altered spectral properties, improved light sensitivity, subcellular targeting, and beyond. Their broadening applications are also explored across neuroscience, cardiovascular biology, hematology, plant sciences, and other emerging fields. In addition, current trends such as all‐optical approaches, multiplexed control, and clinical translation, particularly in vision restoration are discussed. Finally, ongoing challenges are addressed and outline future directions in optogenetic tool development and in vivo applications, positioning optogenetics as a transformative platform for basic research and therapeutic advancement.

## Graphical abstract

Optogenetics provides up to millisecond scale, cell type‐specific control of biology using light‐sensitive proteins. This review chronicles two decades of tool development, from microbial rhodopsins and photoactivated enzymes to allosteric switches, surveys broad applications in neuroscience, molecular and cell biology, cardiology, immunology, and oncology etc., and highlights emerging trends and clinical progress, positioning optogenetics as a transformative platform for basic research and novel therapies.

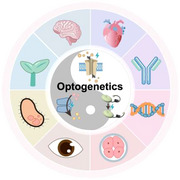

## 1 From photoreceptors to Optogenetics

**Figure 1:**
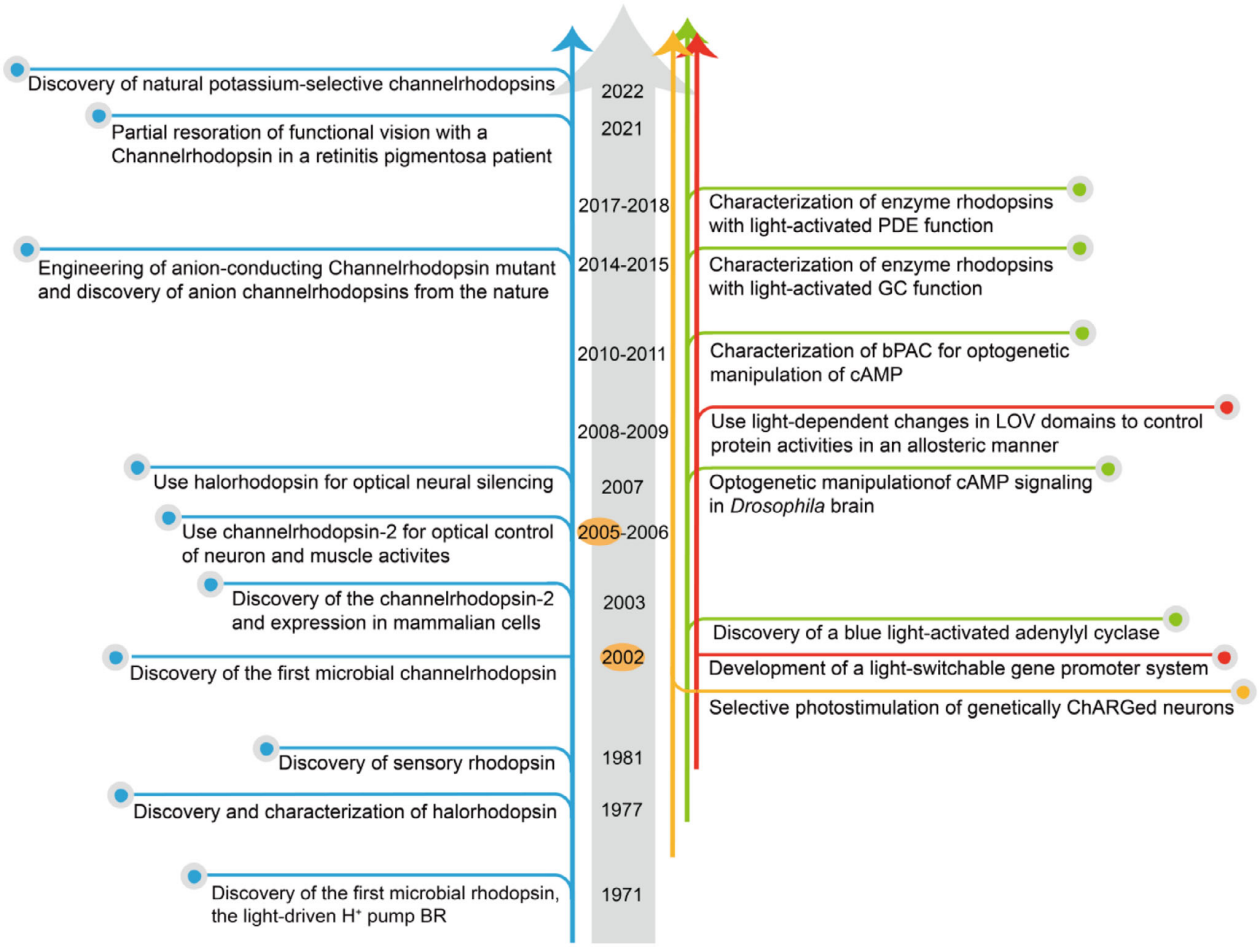
Timeline of selected key advances in the development of optogenetic tools. The left section (blue lines) highlights major discoveries of ion‐transporting microbial rhodopsins and their applications. The right section details significant advancements in optogenetic tools for manipulating cAMP and cGMP (outlined with green lines) and optogenetic switches for controlling gene expression and protein activities (outlined with red lines); the trial with chARGe to stimulate neurons optically is outlined with orange lines. Abbreviations: BR, Bacteriorhodopsin; bPAC, Beggiatoa Photoactivatable Adenylyl Cyclase; LOV, the photoreactive LOV (light oxygen voltage) domain from phototropin; GC, Guanylyl cyclase; PDE, Phosphodiesterase. Due to space limitations, not all optogenetic tools are highlighted in this timeline.

The development of any new technology depends on two fundamental conditions: 1) a clearly defined need and 2) the feasibility of its implementation. The conceptual need for an optogenetics‐like approach was already proposed by Francis Crick in 1979, envisioning the use of light for precise spatiotemporal control of neuronal activity.^[^ ^1^ ^]^ However, at that time, no method existed to render neurons responsive to light. While the breakthrough of optogenetics is most often recognized through its successful application in neuroscience,^[^ ^2^, ^3^, ^4^, ^5^, ^6^, ^7^ ^]^ this achievement was only possible once all foundational components were in place. In particular, the development of optogenetics was deeply rooted in decades of research on natural photoreceptors (**Figure** **1** and **Table** **1**). Figure 1 outlines the evolution of optogenetics, from the 1970s discovery of ion‐transporting microbial rhodopsins and their neuronal deployment in 2005, through the mid 2000s development of light‐activated cyclases for cAMP/cGMP (cyclic adenosine monophosphate/cyclic guanosine monophosphate) control, to the advent of LOV (light‐oxygen‐voltage domain) and Cryptochrome‐based switches around 2010 that permit direct regulation of gene expression and protein activity. While the landmark moment in optogenetics is often marked by its successful application in neuroscience in 2005, it is important to note that most of the foundational components were already in place by 2002. It took approximately three more years for these tools to be effectively applied in neural systems, culminating in the birth of modern optogenetics. The eventual realization of optogenetics was made possible by the convergence of this unmet need and the technological feasibility enabled by photoreceptor biology, leading to its emergence as a transformative tool in modern neuroscience.

**Table 1:** The chromophores and biological functions of well‐characterized photoreceptor families.

Photoreceptor Family	Chromophores	Photochemistry	Biological Function
Rhodopsin	retinal	Cis‐trans isomerization	visual system, phototaxis, Circadian regulation
Phytochrome	phytochromobilin	Cis‐trans isomerization	seed germination, photomorphogenesis, flowering, vernalization, etc.
Xanthopsin	p‐Coumaric acid	Cis‐trans isomerization	Phototactic response, Regulation of flavonoid synthesis
Cryptochrome	Flavin (FAD) and pterin (5,10‐ MTHF)	Photoelectron transfer	Phototropism, Circadian regulation, Photomorphogenesis
LOV proteins	Flavin (FMN)	FMN‐cysteinyl adduct formation	Phototropism, stomata opening, chloroplast movement
BLUF proteins	Flavin (FAD)	Rearrangement of hydrogen bonds	control enzyme activity or gene expression

Abbreviations: FMN (Flavin mononucleotide), FAD (Flavin adenine dinucleotide), 5,10‐MTHF (5,10‐methenyltetrahydrofolic acid).

### 1.1 A Snapshot of Photosensing and Natural Photoreceptors

Sunlight provides two fundamental opportunities for living organisms on earth, as a source of energy and environmental signals. To harness solar energy and perceive environmental cues, many organisms, including plants, bacteria, fungi, and higher eukaryotes, have evolved a variety of native photoreceptor proteins responsive to different wavelengths. These photoreceptors facilitate diverse physiological adaptations to light, enhancing the organisms' ability to thrive in their respective environments.

In 1876, Franz Boll discovered the first photosensory protein rhodopsin (the apoprotein opsin bound to the chromophore retinal), which is the light‐sensitive molecule in the rods of the retina.^[^ ^8^ ^]^ The majority of the biological light sensors consist of a protein/pigment complex that alters the activity of a cognate biological effector upon absorption of a photon. Rhodopsins, particularly those from the animal visual system, known as type II or metazoan rhodopsins, are G‐protein‐coupled receptors. They play a crucial role in sensing light and inducing membrane hyperpolarization through various signaling pathways. Nowadays, numerous photoreceptor proteins have been discovered and categorized into several families.^[^ ^9^ ^]^ The most logical classification method is based on the chemical structure of their light‐absorbing chromophores. Consequently, the most significant families are the rhodopsins, phytochromes, photoactive yellow proteins (PYPs, also referred to as xanthopsins), cryptochromes, phototropins with LOV domains, and blue‐light using flavin (BLUF) proteins.^[^ ^10^ ^]^

Photoreceptors sense light by incorporating an exogenous chromophore, which absorbs light and transmits energy to the protein backbone, leading to conformational changes and receptor activation. As shown in Table 1, the well‐studied types of photosensory proteins primarily utilize mainly four different chromophores.^[^ ^11^ ^]^ Rhodopsins, which detect a broad spectrum of light from UV to red, utilize retinal as their chromophore (Table 1). It is remarkable that the same retinal chromophore in rhodopsins can produce a broad range of absorption maxima, spanning the entire visible light spectrum of 300–700 nm.^[^ ^12^ ^]^ The protonation status of the chromophore and the protein environment, including charged, uncharged, or aromatic amino acids, play crucial roles in color‐tuning.^[^ ^13^, ^14^, ^15^ ^]^ UV‐ and blue‐light‐sensing flavoproteins (≈300–500 nm), such as cryptochromes, phototropins, and BLUF proteins, use flavin mononucleotide (FMN) or flavin adenine dinucleotide (FAD) as their chromophore (Table 1). Xanthopsins, which also sense UV and blue light, incorporate 4‐hydroxy‐cinnamic acid as their chromophore. Phytochromes, primarily sensing far‐red and near‐infrared (NIR) light, use various linear tetrapyrrole bilins as chromophores (Table 1). It is worth noting that the photoreceptors UVR8 (UV resistance locus 8) from plants and algae utilize three intrinsic tryptophan residues (Trp233, 285, and 337) to form a tryptophan‐cluster in the β‐propeller core to facilitate UV‐B perception, thereby becoming activated via a dimer‐to‐monomer switch.^[^ ^16^, ^17^, ^18^ ^]^ Similarly, the recently discovered UV receptor LITE‐1 from *Caenorhabditis elegans* employs a comparable mechanism.^[^ ^19^ ^]^

### 1.2 The Establishment of Optogenetics

Entering the 21st century, the laboratories of Gero Miesenböck and Rich Kramer began implementing photosensitive actuators into host cells.^[^ ^20^, ^21^ ^]^ Although these initial systems were not optimal, being either too complex or too slow in their electrical responses, they played a crucial role in sensitizing neuroscientists to the potential of light‐modulated proteins.

Approximately 100 years after the discovery of animal rhodopsin, microbial rhodopsins were identified in the haloarchaea *Halobacterium salinarum* ^[^ ^22^ ^]^ (Figure 1). The so‐called bacteriorhodopsin (BR) from haloarchaea functions as a light‐driven proton pump. It captures light energy to transport protons across the membrane, out of the cell. The proton‐motive force generated by bacteriorhodopsin is harnessed by ATP synthase to produce adenosine triphosphate (ATP).^[^ ^23^ ^]^ Ion‐pumping rhodopsins enable a diverse array of bacteria and archaea to live photoautotrophically.^[^ ^24^, ^25^, ^26^ ^]^

Clues suggesting the presence of light‐gated ion channels in the green alga *Chlamydomonas reinhardtii* were first reported in 1991.^[^ ^27^ ^]^ Approximately a decade later, microbial rhodopsins with light‐gated ion channel activity from *C. reinhardtii* were successfully identified and functionally characterized^[^ ^28^, ^29^ ^]^ (Figure 1). The light‐gated cation channel, channelrhodopsin‐2 (ChR2), played a pioneering role in the development of optogenetics and has significantly impacted neuroscience as a valuable tool. In the paper on ChR2, Nagel et al. highlighted: “… expression of ChR2 in oocytes or mammalian cells may be used as a powerful tool to increase cytoplasmic Ca^2+^ concentration or to depolarize the cell membrane, simply by illumination.” Shortly thereafter, ChR2 was adopted by several neuroscience researchers who utilized it to manipulate neuronal activity. The seminal publications in 2005 and 2006 from the laboratories of Karl Deisseroth, Alexander Gottschalk, Stefan Herlitze, Hiromu Yawo, and Zhuohua Pan demonstrated the functionality of ChR2 in various systems, including the hippocampal neurons, transgenic worms, the spines of living chicken embryos, PC12 cells, mouse brain slices, and retina of blind mice.^[^ ^3^, ^4^, ^5^, ^6^, ^7^ ^]^ These pioneering studies marked the inception of the field now known as optogenetics, and the name “Optogenetics” gained popularity in 2006 for the light‐induced generation of action potentials in nerve cells, achieved through depolarization via several different approaches.^[^ ^30^ ^]^

Optogenetics is now a simple concept: a heterologously expressed (genetics) photosensitive protein is activated by light (opto), leading to an activity change in target cells or an intact organism. Optogenetics has become a widely adopted technique that leverages genetic engineering and optical technology.^[^ ^31^ ^]^ The application of optogenetics has broadened beyond neuroscience into various research fields, and the optogenetic toolkit has expanded to include photoreceptor families beyond just rhodopsins.^[^ ^32^ ^]^ A brief landscape for the development of optogenetic tools and applications is drawn in Figure 1.

In recent years, optogenetic methods have revolutionized neuroscience and cell biology by enabling precise spatiotemporal control of membrane potential, enzymatic activity, and protein‐protein interactions.^[^ ^33^, ^34^, ^35^ ^]^ For instance, ChRs were used to elucidate neuronal connectivity,^[^ ^36^ ^]^ induce synaptic plasticity^[^ ^37^ ^]^ and modulate behavior in ChR‐expressing animals such as flies, worms, zebrafish, chickens, and rodents.^[^ ^2^, ^38^, ^39^ ^]^ Notably, in 2021, a red‐shifted channelrhodopsin (ChR) was successfully used in a clinical setting to partially restore vision in a blind patient.^[^ ^40^ ^]^

## 2 The Application and Development of Optogenetics

### 2.1 Ion‐Conducting Optogenetic Tools

Microbial rhodopsins exhibit faster kinetics and are easier to genetically modify, making them more commonly used in optogenetic studies.^[^ ^41^ ^]^ When illuminated within a specific wavelength range, the retinal in microbial rhodopsins undergoes isomerization from all‐trans to 13‐cis. This isomerization induces a conformational change in the rhodopsins, subsequently causing channel opening and ionic conductance.^[^ ^42^ ^]^ The versatility of microbial rhodopsins now has enabled their application across a wide range of biological fields.

#### 2.1.1 Cation Channelrhodopsins: Pioneering Tools in Optogenetics

Among microbial rhodopsins, ChR2 isolated from *C. reinhardtii* is the most widely used and has been central to the success of optogenetics. Its ability to induce action potentials in neurons by controlling cation influx under blue light has made it a powerful tool for neuroscience research. ChR2 is a light‐gated cation channel permeable to H^+^, Na^+^, K^+^, and weakly to Ca^2+^. By addressing the limitations of ChR2, including its modest single‐channel conductance, non‐specific ion selectivity, and low Ca^2+^ permeability etc., ChR2 variants have been engineered to fulfill different requirements, such as the first ChR2 H134R mutant^[^ ^43^ ^]^ with higher conductivity, enhanced expression and photocurrent,^[^ ^44^ ^]^ red‐shifted action spectrum for increased tissue penetrability (ReaChR),^[^ ^45^ ^]^ and various ChR variants with increased Ca^2+^ conductance, including the L132C mutation,^[^ ^46^ ^]^ the D156H mutant,^[^ ^47^, ^48^ ^]^ PsCatCh 2.0^[^ ^49^, ^50^ ^]^ and CapChR2.^[^ ^51^ ^]^ In parallel to molecular engineering, newly discovered ChR variants also showed different features. For example, the Chronos (*Stigeoclonium helveticum* ChR, ShChR) showed faster channel closing, which is especially valuable for experiments requiring high temporal resolution. Another natural ChR variant Chrimson (CnChR1 from *Chlamydomonas noctigama*) had a red‐shifted action.^[^ ^9^ ^]^ Red‐shifted rhodopsins are important for in vivo applications, particularly in brain tissues, because red light penetrates deeper into biological tissues than blue light. Red‐shifted spectrum together with enhanced light sensitivity would make an ideal tool for non‐invasive stimulation through intact tissues.

#### 2.1.2 Halorhodopsin: Pioneering Tools for Optogenetic Neuronal Inhibition

While ChRs are primarily used for neuronal activation, Halorhodopsins (HR), such as the NpHR from *Natronomonas pharaonis*, are pioneering tools for silencing neuronal.^[^ ^52^, ^53^ ^]^ HRs function as light‐driven chloride pumps that hyperpolarize neurons by actively transporting chloride ions (Cl^−^) into the cell upon exposure to yellow light. This hyperpolarization inhibits the neuron, preventing it from firing action potentials. The enhanced HR variants, such as eNpHR3.0, exhibited improved membrane trafficking and larger photocurrents, making them more effective for neuronal inhibition.^[^ ^54^ ^]^ Inhibitory tools allow for the investigation of the role of particular neural pathways in behavior and neurological disorders. This capability is particularly valuable for studying neurological disorders such as epilepsy and chronic pain, where overactivity in certain neurons contributes to disease pathology. Another light‐driven chloride pump, Jaws,^[^ ^55^ ^]^ was used to reduce cortical activity and showed potential to stop epileptic seizures. However, the photocycle efficiency of a light‐driven ion pump, such as Jaws is at least 1000 times lower than that of light‐gated ion channel. Constant and intense illumination of ion pumps is required to maintain a hyperpolarized membrane potential, which may cause heating effect and damage after long‐term illumination and is not suitable for long‐term inhibition. In addition, during long‐term stimulation, these pumps will significantly affect ion concentration gradients across the cell membrane.^[^ ^56^, ^57^ ^]^ When the light switches off in neurons hyperpolarized by Jaws, there is a strong post‐illumination rebound, which can even lead to action potential firing in the neurons targeted for silencing.^[^ ^58^ ^]^

#### 2.1.3 Bacteriorhodopsin: Proton Pumps and Channels for pH Manipulation

Bacteriorhodopsin (BR) is a light‐driven proton pump that exports H⁺ to build a proton gradient for ATP synthesis.^[^ ^22^, ^59^, ^60^ ^]^ Early heterologous expression in yeast, frog oocytes, and HEK293 cells demonstrated robust light‐activated proton currents, enabling detailed electrophysiological characterization.^[^ ^61^, ^62^, ^63^ ^]^ Protonpumping rhodopsins can hyperpolarize neurons under light, offering potential early routes to optogenetic silencing,^[^ ^64^ ^]^ but their inhibitory effects are inconsistent, eArch3.0, for example, triggers pH‐dependent Ca^2^⁺ influx and increased vesicle release.^[^ ^65^ ^]^ Convincing application with proton pump rhodopsins will be to manipulate intracellular pH levels. The regulation of intracellular pH or proton flux as a signal messenger was proposed two decades ago.^[^ ^66^, ^67^ ^]^ While extracellular pH can signal microbial behavior, intracellular pH affects enzyme activity and reaction rates, protein stability, and the structure of nucleic acids and many other biological molecules. Theoretically, every macromolecule acts as a “pH sensor”.^[^ ^68^ ^]^ Recent progress in engineering light‐gated proton channels^[^ ^69^, ^70^, ^71^ ^]^ allows bidirectional regulation of pH, efflux via proton pumps and influx via proton channels.

#### 2.1.4 Anion Conducting Channelrhodopsins: More Effective Inhibitory Tools but not Always

The E90R mutation broadened ChR2's conductance to include anions.^[^ ^72^ ^]^ Soon after that, naturally occurring anion‐conducting ChRs (ACRs) were discovered from the cryptophyte alga *Guillardia theta*, GtACR1 and GtACR2,^[^ ^73^ ^]^ allowing for precise neuronal silencing without the need for chloride pumps like NpHR.^[^ ^74^ ^]^ Activation of GtACR1 is roughly equivalent to opening a Cl^−^ conductance, offer a more physiological approach by mimicking endogenous GABA/glycine signaling. Cl^−^‐driven inhibition can be paradoxical: in mature neurons or compartments like axons, inefficient Cl^−^ extrusion leads to high intracellular Cl^−^ and depolarization upon ACR activation.^[^ ^75^, ^76^, ^77^, ^78^ ^]^ Recent advancements, such as the development of soma‐targeted ACRs, have significantly improved neuronal silencing.^[^ ^79^ ^]^ Optogenetic chloride channels can produce ambiguous effects on membrane potential depending on the intracellular Cl^−^ concentration.^[^ ^80^ ^]^ Thus, ACRs require careful consideration regarding their effects in different neuronal subtypes and environments.

#### 2.1.5 Kalium Channelrhodopsins: Light‐Gated K+ Channel as the Long‐Awaited Tool for Inhibition?

The electric excitability of muscle, heart, and brain tissue relies on the precise interplay of Na^+^‐ and K^+^‐selective ion channels. While non‐selective cation‐conducting ChRs are well established for excitation, light‐gated K^+^ channels were thought to be the long‐awaited efficient and versatile optogenetic inhibitors. Synthetic light‐regulated K⁺ channels, BLINK1 and BLINK2, were engineered from a viral K⁺ channel linked to a blue‐light‐sensing LOV2 domain.^[^ ^81^, ^82^ ^]^ The combination of light‐gated nucleotidyl cyclase and cyclic nucleotide‐gated (CNG) K^+^ channels yielded light‐gated K^+^ channels, such as SthK‐bP^[^ ^83^ ^]^ and PAC‐K,^[^ ^84^ ^]^ with large conductance. Natural KCRs were then identified in the stramenopile protist *Hyphochytrium catenoides* (HcKCRs)^[^ ^85^ ^]^ and *Wobblia lunata* (WiChR).^[^ ^86^ ^]^ KCRs are a new class of light‐gated K⁺ channels that hyperpolarize cells via high K⁺ efflux, offering robust inhibition where Cl^−^ based tools may falter. In Drosophila, *C. elegans*, and zebrafish, KCR variants with improved trafficking suppress neural activity and behavior with potency comparable to ACR1 but lower toxicity and better performance at high intracellular Cl^−^ levels.^[^ ^80^ ^]^ In direct comparisons with ACR1, KCR1 has shown comparable potency, but with the added advantages of reduced toxicity and superior performance in cells with high Cl^−^ concentrations. HcKCR1‐hs, a highly sensitive KCR variant, has shown potential for transcranial optogenetic inhibition in rodent epilepsy models.^[^ ^87^ ^]^ In the model organism *C. elegans*, stimulation of HcKCR1 in cholinergic neurons induced inhibition and muscle relaxation under optimized light conditions.^[^ ^88^ ^]^ However, long‐term and repeated stimulation can induce activation drift. Recently it was found that stabilized ion‐selectivity mutations in KCRs could correct drift and maintain consistent inhibition over extended protocols, further improving tool reliability.^[^ ^89^ ^]^

Microbial rhodopsins comprise a diverse and extensively studied family of light‐gated proteins in optogenetics; for comprehensive reviews of their kinetics, ion selectivity, and tissue penetration characteristics, see^[^ ^35^, ^90^, ^91^ ^]^

### 2.2 Optogenetic Manipulation of cAMP and cGMP

#### 2.2.1 PAC: Photoactivated Adenylyl Cyclase

cAMP is a major second messenger, which plays pivotal roles in cell signaling and is involved in both physiological and pathological processes such as synaptic plasticity, which is key for learning and memory processes.^[^ ^92^ ^]^ Optogenetic manipulation of cAMP offers targeted, dynamic control with light, capabilities beyond those of traditional pharmacological methods. In 2002, the first photoactivated adenylyl cyclase (PAC) was identified from the unicellular flagellate *Euglena gracilis* and named EuPAC, which is capable of sensing light and showing phototaxis and photophobic response (Figure 1).^[^ ^93^ ^]^ Optogenetic manipulation of cAMP was then first accomplished by expressing the EuPAC in the brain of *Drosophila melanogaster*, where its neuronal expression led to light‐induced behavioral changes^[^ ^94^ ^]^ (Figure 1). The bacterium *Beggiatoa* (bPAC), a minimal BLUF–adenylyl cyclase fusion, offers low dark activity, high light responsiveness, and compact size, making it an ideal optogenetic tool for precise cAMP control.^[^ ^95^ ^]^ Applications with bPAC include rescuing infertility,^[^ ^96^ ^]^ controlling insulin release,^[^ ^97^ ^]^ neuronal repair,^[^ ^98^ ^]^ and modulating the release of neurotransmitters^[^ ^99^ ^]^ and synaptic plasticity.^[^ ^100^, ^101^ ^]^ Recently discovered biPAC^[^ ^102^ ^]^ and a membrane‐anchored bPAC F198Y mutant PACmn^[^ ^103^ ^]^ further minimized dark activity, enabling rapid, reversible cAMP/PKA activation in neurons.

Beyond bluelight PACs, red and NIR–responsive versions have been engineered using bacterial phytochrome and cyanobacteriochrome sensors to improve tissue penetration. For example, a cyanobacteriochrome PAC (cPAC) from *Microcoleus* sp. PCC 7113 features a bistable photocycle, blue light activates and green light inactivates its cyclase, and it can be tuned to NIR wavelengths.^[^ ^104^ ^]^ Etzl et al. fused *Deinococcus radiodurans* bacteriophytochrome with *Synechocystis* cyclase domains to create red‐light‐regulated guanylate/adenylate cyclases with low dark activity and high dynamic range, effectively modulating *C. elegans* locomotion.^[^ ^105^ ^]^ Xu et al. further advanced this approach by swapping photosensory components to engineer DmPAC, a red‐responsive PAC with a 40‐fold activity boost under red light, outperforming previous variants.^[^ ^106^ ^]^ In addition, red‐light‐regulated diguanylate cyclases (DGCs) fused to bacteriophytochrome domains have been developed to optically control c‐di‐GMP levels,^[^ ^107^ ^]^ though their dynamic range is modest, typically around a 10‐fold increase upon illumination. By contrast, blue‐light‐activated PACs can achieve over 1000‐fold activation, offering far greater dynamic responsiveness.^[^ ^103^ ^]^

Pilot experiments have begun to manipulate cAMP signaling at the subcellular level, such as targeting bPAC to synaptic vesicles^[^ ^108^, ^109^, ^110^ ^]^ and cilia.^[^ ^111^ ^]^ Additional genetic tools have been developed to modulate and study cAMP signaling. For example, the cAMP sponge sequesters cAMP to reduce its local concentration,^[^ ^112^ ^]^ while various genetically encoded sensors enable real‐time visualization of cAMP dynamics.^[^ ^113^ ^]^ The optogenetic toolkit has also expanded to downstream effectors such as PKA; Yi et al. engineered a photoactivatable PKA inhibitor (PA‐PKI) using the light‐sensitive LOV domain from plant phototropin to achieve light‐controlled inhibition of PKA activity.^[^ ^114^ ^]^

#### 2.2.2 Cyclase Rhodopsins: Light‐Gated GC

As key second messengers, cAMP and cGMP govern vital cellular processes. A light‐activated guanylyl cyclase, BIgC (or bPGC), was first engineered from bPAC by mutating its cyclase domain based on GC–AC homology. However, bPGC exhibited limited light responsiveness and retained residual adenylyl cyclase activity, limiting its specificity and effectiveness.^[^ ^115^ ^]^ A novel gene encoding a type I rhodopsin fused to a guanylyl cyclase domain, BeGC1, was later discovered in *Blastocladiella emersonii* and shown to mediate green light–induced phototaxis via cGMP production in zoospores.^[^ ^116^ ^]^ This naturally occurring guanylyl cyclase was later developed into BeCyclOp (also known as beRhGC), a green light–activated optogenetic tool with an exceptional light/dark (L/D) activity ratio >5 000 and high cGMP specificity.^[^ ^117^, ^118^ ^]^ Unlike typical seven‐transmembrane rhodopsins, BeCyclOp possesses an unusual eight‐transmembrane topology with a cytosolic N‐terminus.^[^ ^118^ ^]^ Targeted mutations in its cyclase domain enabled substrate switching, yielding red light‐activated rhodopsin adenylyl cyclases (RhACs) with an L/D ratio of ≈280, outperforming previous red light‐activated ACs.^[^ ^119^ ^]^

Additionally, various cyclase rhodopsins have been identified in green algae. For example, 2c‐Cyclops are light‐inhibited, ATP‐dependent guanylyl cyclases.^[^ ^120^ ^]^ The engineered switch‐Cyclop exhibits bistable behavior, activated by a brief 380 nm UV light pulse and deactivated by blue or green light.^[^ ^121^ ^]^ Near infrared–sensitive rhodopsins like NeoR offer potential promise for deep‐tissue applications, although their light‐regulated cyclase activity remains limited.^[^ ^122^ ^]^

#### 2.2.3 Light‐Activated Phosphodiesterases: Valuable Tools Awaiting Optimization

Furthermore, photoactivated PDEs are highly desired to counteract the role of endogenous cAMP and cGMP levels, which will enable bidirectional manipulation of these second messengers. Engineered light‐activated phosphodiesterases (LAPD)^[^ ^123^ ^]^ and natural Rhodopsin Phosphodiesterase (RhoPDE)^[^ ^124^, ^125^, ^126^, ^127^, ^128^, ^129^ ^]^ have been used to hydrolyze cAMP and cGMP. However, these tools tend to exhibit relatively high dark activity and limited dynamic activation range, which restricts their effectiveness in many applications. Currently, the L/D activity ratios of RhoPDEs from choanoflagellates^[^ ^125^, ^130^ ^]^ are low and they are more specific to degrade cGMP. Further optimization is required to improve their dynamic range for more effective manipulation of cAMP and cGMP signaling.

### 2.3 Light‐Induced Molecular Interaction or Light‐Regulated Synthetic Molecular Devices

The success of microbial rhodopsins and PACs as optogenetic tools generated interest in further possibilities to regulate cells by light. Thus, other natural light‐activated proteins were established as tools for optogenetic applications, and more new areas of general cell biology and molecular biology are also being made regulatable by light. Developed from microbial and plant photoreceptors, such light‐regulated synthetic molecular devices rely on a change in protein conformation or enzymatic activity after photon absorption.^[^ ^131^ ^]^ This group of optogenetic tools allows researchers to precisely control protein‐protein interactions, subcellular localization, gene expression and protein activity using light. Early in 2002, a red light gene‐expression switch was introduced in yeast cells^[^ ^132^ ^]^ (Figure 1).

In 2009, Wu et al. developed a new approach to produce genetically encoded photoactivatable derivatives of Rac1, a key GTPase regulating actin cytoskeletal dynamics in metazoan cells. They made use of the light‐induced conformation change of the photoreactive LOV (light oxygen voltage) domain from the plant photoreceptor phototropin, which blocks Rac1 interactions until illumination released the Jα helix linking LOV to Rac1. Photoactivatable Rac1 (PA‐Rac1) could be reversibly activated by blue light to generate precisely localized cell protrusions and ruffling.^[^ ^133^ ^]^

Based on the same photoreactive domain, Guntas et al. developed a light‐induced dimerization system iLID.^[^ ^134^ ^]^ The iLID was designed by incorporating the LOV‐Jα domain with a naturally binding pair from Escherichia coli: seven amino acids of SsrA peptide and its binding partner SspB, a 13‐kDa adaptor protein.^[^ ^135^ ^]^ In the dark, the SsrA peptide, fused to LOV‐Jα, is sterically blocked from binding SspB. When illuminated with blue light, the C‐terminal Jα helix of the LOV2 domain undocks from the protein, allowing the SsrA peptide to bind SspB. The iLID system was used for optogenetic manipulation of different cells by light‐driven protein dimerization or protein localization to specific subcellular domains.^[^ ^136^, ^137^ ^]^ Combining the iLID system with biological membranes to anchor proteins, the mem‐iLID was used to fast and flexible protein purification with light.^[^ ^138^ ^]^

Other light‐gated protein dimerization systems have been developed utilizing natural photoreceptors like cryptochrome,^[^ ^139^ ^]^ phytochrome,^[^ ^132^ ^]^ and others., and applied in the optogenetics field.^[^ ^140^, ^141^ ^]^ Another method to control the protein activity was to use the photo‐induced protein dissociation system by light‐induced releasing of the active site, which is cadged in the dark. The Dronpa system was also applied to regulate GTPase and protein kinase activity. The LOVTRAP as a photo‐induced dissociation system could also be potentially used to regulate protein. Recently, another LOV domain‐containing photoreceptor, BcLOV4, from the fungus *Botrytis cinerea* showed direct light‐regulated binding to anionic membrane phospholipids.^[^ ^142^ ^]^ The single‐component optogenetic translocation tool was used for spatiotemporally precise induction of Rho‐family GTPase signaling at the GEF or GTPase level.^[^ ^143^ ^]^

The field has quickly advanced, yielding hundreds of applications in microbial, yeast, plant and animal systems.^[^ ^144^ ^]^ These systems enable non‐invasive, reversible, and highly specific manipulation of various cellular processes beyond ion channel control, which is in turn used to control a wide range of cellular processes.^[^ ^145^, ^146^ ^]^ For a comprehensive list and detailed characteristics of nonrhodopsin optogenetic switches, see OptoBase (www.optobase.org).

### 2.4 Optogenetics Expanded

**Figure 2:**
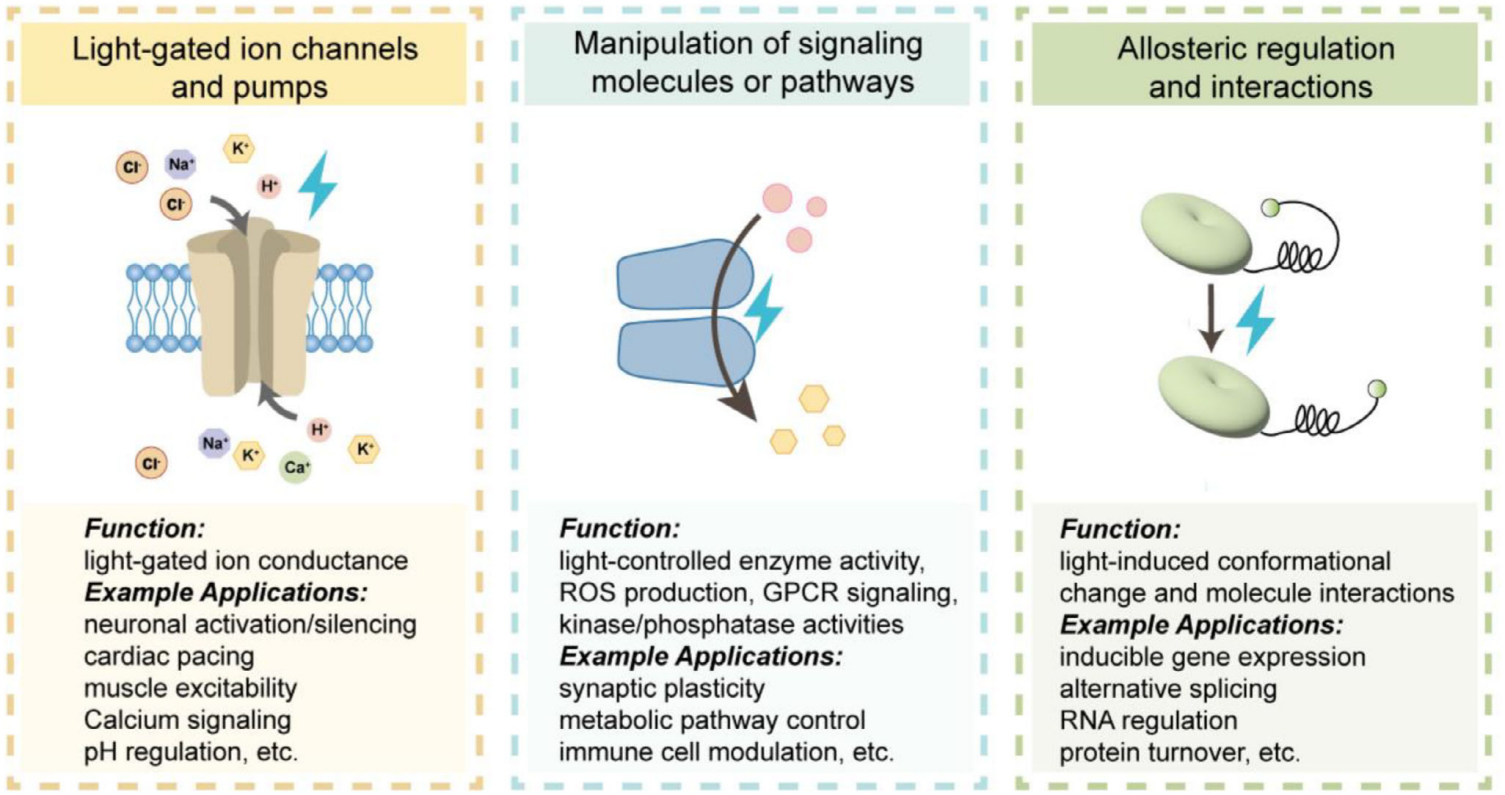
General classification of optogenetic tools. Optogenetic actuators can be broadly categorized into three major functional groups: 1) light‐gated ion channels and pumps for controlling membrane potential and ion homeostasis, 2) tools for manipulating intracellular signaling pathways, such as cAMP/cGMP levels or GPCR cascades, and 3) systems enabling light‐regulated protein‐protein interactions, allosteric regulation, and gene expression.

With the advancement of optical technologies, light has become an increasingly powerful tool in biomedical research, offering exceptional spatial and temporal precision. While initially used primarily for monitoring and observation, light is now widely employed for active manipulation of biological processes through optogenetic tools and light‐responsive molecules. As shown in **Figure** **2**, these optogenetic systems extend far beyond earlier applications and can generally be categorized into three major classes: 1) light‐gated ion channels and pumps, 2) modulators of intracellular signaling pathways and second messengers, and 3) tools for controlling allosteric regulation and protein‐protein interactions (Figure 2). These classes reflect the expanding scope of optogenetics beyond neural excitation, enabling precise control over a wide range of cellular and molecular processes.

A more comprehensive, though not exhaustive, overview of current optogenetic tools is provided in **Table** **2**.

**Table 2:** A brief summary of existing optogenetic tools.

	Function	Example Tools	Applications
**Light‐gated ion channels and pumps**	cation ChRs	ChR^[^ ^28^ ^]^ homologs and variants, summarized in recent reviews^[^ ^90^, ^144^ ^]^	depolarize cells, activate neurons.
	anion ChRs	GtACR1, GtACR2,^[^ ^73^ ^]^ ZipACR,^[^ ^147^ ^]^ MerMAIDs^[^ ^148^ ^]^	hyperpolarize or depolarize cells, inhibit (or activate in rare cases) neurons^[^ ^79^ ^]^
	potassium Channels	BLINK,^[^ ^81^, ^82^ ^]^ SthK‐bP,^[^ ^83^ ^]^ PAC‐K,^[^ ^84^ ^]^ HcKCR1,^[^ ^85^ ^]^ WiChR^[^ ^86^ ^]^	silence neurons,^[^ ^83^, ^84^, ^85^ ^]^ pain relief^[^ ^82^ ^]^
	Ca^2+^‐conductive channels	OLF‐bP,^[^ ^83^ ^]^ CatCh,^[^ ^46^ ^]^ ChR2‐XXM,^[^ ^48^ ^]^ PsCatCh 2.0,^[^ ^50^ ^]^ CapChR^[^ ^51^ ^]^	manipulate Ca^2+^ signaling
	pH manipulation	Proton pump rhodopsins and their channel mutants,^[^ ^69^, ^71^, ^149^ ^]^ H^+^‐conductive ChRs^[^ ^29^, ^150^, ^151^ ^]^ …	regulate pH and proton signaling^[^ ^152^, ^153^ ^]^
**Manipulation of signaling molecules or pathways**	adenylyl cyclase	bPAC,^[^ ^95^, ^103^, ^115^ ^]^ CaRhAC,^[^ ^119^ ^]^ PACmn,^[^ ^103^ ^]^ biPAC^[^ ^102^ ^]^	manipulate cAMP signaling
	guanylyl cyclase	CyclOp, also known as RhGC^[^ ^117^ ^]^	manipulate cGMP signaling
	OptoGPCR^[^ ^154^, ^155^, ^156^ ^]^	ChARGe,^[^ ^20^ ^]^ optoDopR,^[^ ^157^ ^]^ *Pd*CO^[^ ^158^ ^]^	Utilize natural or engineered light‐regulated GPCRs to control G protein signaling pathway.
	production of reactive oxygen species (ROS)	KillerRed,^[^ ^159^ ^]^ SuperNova,^[^ ^160^, ^161^ ^]^ miniSOG^[^ ^162^ ^]^ and miniSOG2^[^ ^163^ ^]^	induce cell ablation,^[^ ^162^, ^163^ ^]^ targeted protein degradation,^[^ ^161^ ^]^ modulating of channel activities^[^ ^164^ ^]^
**Allosteric regulation and interactions**	allosteric regulation	LOV‐Jα based tools such as PA‐Rac1,^[^ ^133^ ^]^ OptoNBs,^[^ ^165^ ^]^ BcLOV4^[^ ^166^ ^]^	regulate the activity of small proteins,^[^ ^167^ ^]^ target endogenous proteins^[^ ^165^ ^]^
	intermolecular interactions	CRY2/CIB1,^[^ ^139^ ^]^ PhyB/PIF,^[^ ^132^, ^168^, ^169^, ^170^ ^]^ iLID^[^ ^134^ ^]^	target endogenous proteins,^[^ ^171^ ^]^ regulate phase separation process,^[^ ^172^ ^]^ protein purification,^[^ ^138^, ^173^ ^]^ manipulate protein activities^[^ ^174^ ^]^
	inducible gene expression	PHYB and PIF6,^[^ ^175^ ^]^ CarH,^[^ ^176^ ^]^ OCPs,^[^ ^177^ ^]^ PULSE^[^ ^178^ ^]^	induce gene expression by light

This table is generated with recent updates according to information from^[^ ^179^ ^]^ under the terms of a creative commons attribution 4.0 international license, copyright 2023 by the author(s), Annual Reviews.

#### 2.4.1 OptoGPCRs: Optogenetic G‐Protein‐Coupled Receptors

OptoGPCRs are a class of light‐sensitive, native or engineered receptors that enable precise control of intracellular signaling pathways using light.^[^ ^156^ ^]^ Early in 2002, the lab of Gero Miesenböck began implementing photosensitive GPCRs into host cells.^[^ ^20^ ^]^ GPCRs are naturally occurring membrane receptors that mediate various physiological processes, including neurotransmission, hormone responses, and immune reactions, by triggering intracellular signaling cascades. OptoGPCRs leverage this native function, but they are modified to be activated by specific wavelengths of light, providing high spatial and temporal control over GPCR signaling. OptoGPCRs provide a powerful tool for studying GPCR‐mediated signaling with high precision, making them invaluable in research areas such as cell signaling, pharmacology, and synthetic biology. In addition to engineered optoGPCRs, the use of natural light‐gated GPCRs, such as animal rhodopsins, is becoming increasingly popular.^[^ ^158^, ^180^ ^]^

#### 2.4.2 Light‐Induced ROS and Light‐Gated Protein Degradation

miniSOG was first developed as a fluorescence tag for correlated light and electron microscopy. It was then used for photoablation of cells by mitochondrial‐targeted miniSOG (mito‐miniSOG) in *C. elegans* neurons because of light‐induced singlet oxygen production.^[^ ^162^ ^]^ Upon blue‐light illumination, mito‐miniSOG causes rapid and effective death of neurons in a cell‐autonomous manner without detectable damage to surrounding tissues. An improved version miniSOG2 was recently reported.^[^ ^163^ ^]^ Fluorescence proteins like KillerRed and SuperNova^[^ ^160^ ^]^ were used in a similar way to induce cell ablation,^[^ ^162^, ^163^ ^]^ targeted degradation of proteins,^[^ ^161^ ^]^ and modulating activities of channels such as TRPA1 and TRPV1^[^ ^164^ ^]^ in animal cells. In a notable example, Goto et al. employed SuperNova, a monomeric red fluorescent protein‐derived photosensitizer, to induce targeted oxidative damage and functional inactivation of synaptic proteins with precise spatial and temporal control.^[^ ^161^ ^]^

Worth noting, Chen et al. developed POT, an optogenetics‐based system for targeted degradation of endogenous proteins that operate independently of ROS. By fusing the protein of interest with Cryptochrome 2 and employing blue light to recruit ubiquitin ligases, this system enables precise, reversible, and efficient degradation of proteins in living cells.^[^ ^181^ ^]^ In a complementary approach, Tague et al. developed LOVdeg, a light‐inducible protein degradation system for *E. coli*, based on a blue light‐sensitive LOV domain. By fusing this tag to target proteins, they achieved rapid and reversible control of protein levels in bacterial cells, demonstrating a versatile tool for dynamic regulation of gene circuits and protein function in prokaryotic systems.^[^ ^182^ ^]^ In addition to genetically encoded systems, photocaged molecules have also been used to induce target protein degradation,^[^ ^183^, ^184^ ^]^ though this topic is not covered in detail here but is discussed in another review.^[^ ^185^ ^]^

#### 2.4.3 All‐Optical Approach: Simultaneous Monitoring and Manipulation of Neural Activity using Multi‐Color Light

All‐optical methods combine genetically encoded activity sensors with optogenetic actuators, enabling simultaneous readout and manipulation of neuronal activity with single‐neuron and single‐spike precision.^[^ ^186^ ^]^ By coexpressing both sensors, such as GCaMP for calcium imaging, and actuators like ChR2 within the same neurons, researchers can control neural circuits in real‐time.^[^ ^187^ ^]^ This approach allows for detailed investigations into how specific neurons contribute to circuit dynamics, behavior, and cognition.

All‐optical methods have also advanced the high‐throughput functional and pharmacological screening of voltage‐gated ion channels. Zhang et al. demonstrated a fully optical electrophysiology system using genetically encoded voltage indicators and optogenetic actuators to probe ion channel kinetics and drug effects with high spatiotemporal resolution.^[^ ^188^ ^]^ In parallel, Klimas et al. introduced OptoDyCE, an automated all‐optical platform tailored for dynamic cardiac electrophysiology screening in human cardiomyocytes.^[^ ^189^ ^]^ Streit and Kleinlogel developed a dynamic all‐optical platform for drug screening targeting cardiac voltage‐gated ion channels.^[^ ^190^ ^]^ One major advance was recently made by Wong et al., who developed an optogenetic drug discovery platform based on cryptochrome CRY2 to induce clustering of PKR and activate the integrated stress response (ISR). This approach enabled high‐throughput screening of over 370000 compounds, leading to the identification of ISR modulators with broad antiviral activity and minimal cytotoxicity. Their work exemplifies how optogenetic control can power therapeutic discovery far beyond traditional neural applications.^[^ ^191^ ^]^ Together, these studies showcase the potential of optogenetics and all‐optical methods for scalable, non‐invasive **drug screening** and ion channel characterization.

All‐optical methods offer precise control over neural activity while allowing for closed‐loop experiments in which neural responses guide real‐time manipulation. However, challenges such as reducing light crosstalk and enhancing coexpression of components remain, but advancements in optical technologies are continually improving spatial and temporal precision. All‐optical approach opens new avenues for studying brain function and developing neurotechnological applications.

## 3 Emerging Trends in Optogenetics

### 3.1 Ion Selectivity and Precision

Optogenetics has emerged as a transformative technology since its inception in neuroscience, enabling precise control of cellular activities using light. Initially, ChRs were discovered as non‐selective cation channels in eukaryotic algae, allowing ions like Na⁺, K⁺, H⁺, and Ca^2^⁺ to pass through cell membranes. This ion flow led to membrane depolarization and the initiation of action potentials in neurons, providing a powerful tool for manipulating neural circuits with high spatiotemporal precision.

As the field evolved, the need for more ion‐selective optogenetic tools to achieve precise control over cellular activities increased and led to the development of ChRs with specific conductance properties, such as anion ChRs, potassium‐conducting ChRs and etc. The engineering of ChRs with enhanced ion specificity offers a pathway toward fine‐tuned inhibition or activation, expanding optogenetic applications across various biological systems.

The refinement of ion specificity in optogenetic tools allows for targeted manipulation of not only neural circuits but also cell signaling pathways and metabolic processes. For instance, the development of Ca^2^⁺‐permeable ChR variants has provided significant advantages in studying Ca^2^⁺ signaling. However, achieving exclusive ion conductance remains challenging. Even with enhanced Ca^2^⁺ conductance, ChR variants like ChR2 XXM2.0 are also permeable to other cations such as Na⁺. Under physiological conditions, where extracellular Na⁺ concentrations are higher than Ca^2^⁺, Na⁺ conductance cannot be entirely excluded. Therefore, ongoing efforts aim to engineer optogenetic tools with even greater ion selectivity and predictability. The KCRs also showed mild Na⁺ conductance in addition to its dominant K^+^ conductance.^[^ ^80^, ^85^, ^86^, ^192^ ^]^ While KCR1 was initially considered a promising inhibitory tool for neuroscience, its tendency to alternate between inhibition and activation depending on the cellular context highlighted the complexity of achieving predictable functions,^[^ ^88^ ^]^ which required further investigation

### 3.2 In Vivo Application and Subcellular Targeting

Advancements in optogenetics are also focusing on transitioning toward more in vivo applications, ensuring that these tools function efficiently in live organisms. This need has driven the development of red‐shifted and highly sensitive optogenetic tools, which are more effective at penetrating tissue due to their sensitivity to longer wavelengths of light. These tools with enhanced deep tissue penetration allowed researchers to target specific cell types or tissue regions more precisely in real‐time studies of neural circuits, cardiac functions, and hormonal signaling.^[^ ^193^ ^]^

However, limitations persist, particularly in the use of retinal as the chromophore for optogenetic tools,^[^ ^122^ ^]^ especially in larger animal models.^[^ ^194^ ^]^ To address these challenges, there has been a push to develop more efficient light delivery systems, including implantable micro‐LEDs (µLED) for localized illumination in animal tissues,^[^ ^195^ ^]^ and upconversion nanoparticles (UCNPs), which can convert NIR light into visible wavelengths, enhancing deep brain stimulation without damaging tissues.^[^ ^196^ ^]^

Achieving subcellular precision is another frontier in optogenetics, particularly when studying second messengers like cAMP and cGMP, which function in highly localized cellular “hot spots”.^[^ ^197^ ^]^ By targeting optogenetic tools to subcellular compartments like the endoplasmic reticulum, mitochondria, or synaptic terminals, researchers can manipulate these signaling events with high spatiotemporal resolution.

A notable innovation is the development of OptoNBs (Optogenetic Nanobodies), a versatile class of photoswitchable proteins capable of light‐controlled binding to untagged target proteins, providing unprecedented subcellular precision.^[^ ^165^ ^]^ These OptoNBs allow for fine‐tuned control of protein interactions and signaling pathways and represent a significant advancement in optogenetic tools for cellular signaling and intracellular interactions across various research fields.

This continued development of precise, tissue‐penetrating, and subcellular‐targeted optogenetic tools is expected to expand the potential of optogenetics in both basic research and clinical applications, enabling deeper insights into complex biological systems.

### 3.3 Light‐Controlled Splicing and RNA Regulation

Beyond protein‐level control, optogenetic strategies have been extended to modulate diverse RNA functions, including splicing, stability, translation, and localization. One approach enables reversible regulation of RNA activity through light‐sensitive RNA‐protein interactions, as shown by an optoribogenetic system responsive to blue light.^[^ ^198^ ^]^ Light‐controllable RNA–protein devices have also been developed to achieve dynamic translational control of synthetic mRNAs in mammalian cells.^[^ ^199^ ^]^ Another method uses engineered RNA‐binding proteins that switch conformation in response to light, offering precise spatiotemporal control of RNA metabolism in living cells.^[^ ^200^ ^]^ In prokaryotic systems, light‐inducible RNA switches have been designed to control gene expression at the mRNA level with high temporal resolution.^[^ ^201^ ^]^ Optogenetic gene editing has also been demonstrated in vivo by coupling light‐triggered gRNA expression to CRISPR activation, enabling targeted manipulation in T lymphocytes.^[^ ^202^ ^]^ Additionally, a system for light‐controlled storage and release of proteins and mRNAs has enabled precise, reversible gene expression in both cultured cells and animals.^[^ ^203^ ^]^

### 3.4 Applications Across Diverse Research Fields

Neuroscience remains at the forefront of optogenetic applications. By using ChRs to induce neuronal excitation and tools like ACRs and KCRs for inhibition, researchers have gained groundbreaking insights into memory, emotion, and neuropsychiatric disorders such as Parkinson's disease and epilepsy.^[^ ^33^, ^35^, ^40^, ^204^, ^205^ ^]^ ChRs also showed potential for repairing of hearing impairment.^[^ ^206^, ^207^ ^]^ In cardiovascular research, optogenetics has enabled non‐invasive control of cardiomyocytes using rhodopsins. This has opened new methods to study heart rhythm disorders like arrhythmias, offering potential possibilities for developing treatments for heart failure and sudden cardiac arrest.^[^ ^35^, ^205^, ^208^, ^209^ ^]^

In cell biology and molecular signaling, optogenetics has been used to control second messenger systems such as cAMP and cGMP. Light‐activated adenylyl and guanylyl cyclases enable manipulation of cellular signaling with unparalleled temporal precision. Additionally, light‐induced molecular interactions using tools like CRY2/CIB1 and iLID allow for precise regulation of protein‐protein interactions and gene expression, extending optogenetics from ion flux modulation to intricate control of cellular machinery. Recent advances have demonstrated the potential of combining optogenetics with CAR T cell therapy to achieve safer and more precise cancer immunotherapy. Huang et al. first engineered light‐controllable CAR T cells using blue light‐sensitive protein domains (such as CRY2/CIB1) to reversibly assemble functional CAR complexes, allowing spatiotemporal control over T cell activation in vitro and in vivo.^[^ ^210^ ^]^ Nguyen et al. (2021) developed a nano‐optogenetic system in which split CAR constructs are activated by upconversion nanoparticles that convert NIR light into blue light, enabling deep tissue activation and minimizing off‐tumor toxicity.^[^ ^211^ ^]^ These studies underscore the emerging role of optogenetics in refining adoptive cell therapies and advancing next‐generation precision immunotherapies.^[^ ^212^ ^]^ In addition to immunotherapy, optogenetics has been increasingly integrated into cancer research to enable precise manipulation of oncogenic signaling and cell death pathways. Light‐controlled hyperactivation of the PI3K pathway has been used to model drug resistance mechanisms in tumor cells, providing dynamic insights into therapy evasion under controlled stimulation conditions.^[^ ^213^ ^]^ Similarly, photoactivatable oncolytic viruses have been engineered to replicate selectively in response to light, enhancing tumor specificity and reducing off‐target effects during virotherapy.^[^ ^214^ ^]^ Furthermore, optogenetic platforms have been developed to induce non‐apoptotic cell death through light‐triggered clustering of death effectors, offering an alternative approach to eliminate cancer cells with high precision.^[^ ^215^ ^]^ These strategies demonstrate the versatility of optogenetics in advancing cancer biology and treatment modalities.

Optogenetic control of protein interactions allows for exploration of molecular pathways involved in cell division, gene expression, and metabolic control, which are universal for most cell types.^[^ ^146^ ^]^ Optogenetics is also gaining traction in biological systems such as metabolism, gut microbiota, and developmental biology. In metabolic research, light‐controlled activation of non‐canonical thermogenic pathways in adipose tissue has been shown to protect against obesity, offering new directions for precise, non‐invasive interventions in energy balance regulation.^[^ ^216^ ^]^ In microbiome studies, optogenetic tools have been applied to gut‐residing bacteria to manipulate metabolic activity in real time, revealing links between microbial function and host longevity in live animals.^[^ ^217^ ^]^ Moreover, in developmental biology, a light‐inducible cell reprogramming system has been developed to modulate gene expression and drive cell fate decisions both in vitro and in chimeric mouse models, demonstrating the feasibility of using optogenetics for dynamic control of tissue development and regeneration.^[^ ^218^ ^]^ These examples highlight the expanding scope of optogenetics beyond neuroscience, with increasing potential in systemic physiology and regenerative medicine.

**Figure 3:**
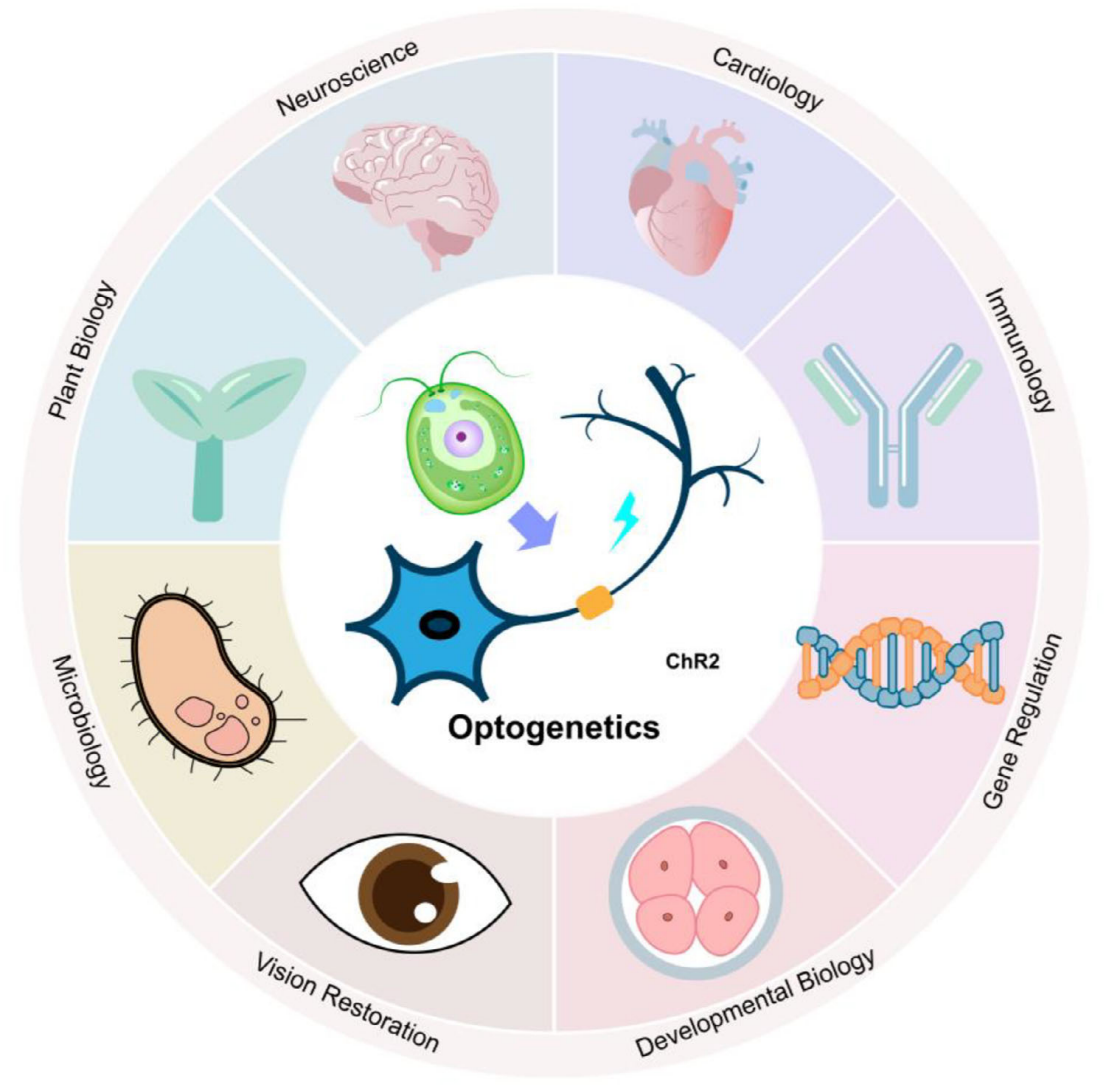
Expanding applications of optogenetics across diverse research fields.Optogenetics, while originally rooted in neuroscience, has rapidly evolved into a versatile platform now applied across diverse fields. Its ability to precisely manipulate biological activity with high spatial and temporal resolution continues to drive innovation in both fundamental research and emerging therapeutic strategies.

Beyond its well‐established role in neuroscience, optogenetics is rapidly extending into a variety of other disciplines. As shown in **Figure** **3**, optogenetics applications span neuroscience, cardiology, immunology, metabolism, plant biology, development, and oncology, leveraging light for millisecondscale, celltype–specific control in both research and emerging therapies. This broad utility underscores optogenetics’ unique combination of spatial, temporal, and molecular specificity.

Hematology represents an emerging frontier for optogenetics. Tools such as BeCyclOp and the high Ca^2^⁺‐conductance ChR2 variant XXM2.0 have been used to study cGMP signaling and Ca^2^⁺ dynamics in megakaryocytes and platelets,^[^ ^219^ ^]^ offering innovative strategies to modulate blood cell behavior. These approaches hold promises for advancing our understanding of clotting disorders and thrombosis. In plant biology, optogenetics has leveraged plants’ natural light responsiveness to modulate intracellular signaling. For instance, ChR2 XXM2.0 was employed to trigger defense responses by elevating cytosolic Ca^2^⁺ levels and promoting ROS production.^[^ ^220^ ^]^ Additionally, optogenetic actuators have been successfully applied to control gene expression, guide pollen tube growth, regulate stomatal opening, and influence overall plant development.^[^ ^74^, ^179^ ^]^

## 4 Future Directions and Clinical Applications

The future of optogenetics will likely be shaped by the development of more precise and diverse tools, expanding the technology's applications across both research and clinical fields. Key directions include:

### 4.1 More Precise and Diverse Tools to be Combined and Targeted to Subcellular Structures

The current optogenetic tools can perform functions in a limited range. Molecular engineering or screening of natural photoreceptors will enrich the tool kit. For example, advancements in engineering optogenetic actuators with enhanced ion specificity will allow for more precise control over cellular activities. This increased specificity will reduce off‐target effects and improve the accuracy and predictability of experiments. Such developments will lead to greater efficiency in both fundamental research and therapeutic applications. The development of tools with different functions and responsive to different wavelengths of light will enable the simultaneous control of multiple cellular processes, such as bidirectional dual‐color control of neurons.^[^ ^221^ ^]^ In one recent trial, multiple ChRs were combined to control the water flux, demonstrating effective light‐controlled water transport via ion fluxes in *X. laevis* oocytes.^[^ ^222^ ^]^ Researchers will increasingly focus on targeting specific subcellular compartments such as the ER, mitochondria, and synaptic terminals. This approach will allow for a finer dissection of localized signaling events with high spatiotemporal resolution. It will be particularly useful in studying second messengers like cAMP, cGMP and Ca^2+^, which function in confined subcellular domains, offering more granular insights into cellular behavior.

### 4.2 Multiplexing Techniques

The optogenetic method can also be or need to be well combined with other methods. Optogenetics has been effectively integrated with CRISPR technologies to enable precise, light‐controlled genome and epigenome manipulation. Light‐inducible CRISPR–Cas systems allow spatiotemporal control of gene editing and transcriptional regulation. Lan et al. reviewed photoactivatable CRISPR constructs for light‐tuned gene expression and synthetic gene circuits,^[^ ^223^ ^]^ while Mathony et al. reviewed optogenetic CRISPR platforms for spatially restricted editing, timed gene activation, and reversible chromatin modulation.^[^ ^224^ ^]^ Optogenetics and chemogenetics represent two powerful but distinct modalities for controlling cellular activity. Optogenetics offers millisecondscale control and subcellular spatial precision by directly modulating light‐sensitive proteins, whereas chemogenetics relies on engineered receptors activated by administered ligands, yielding broader tissue coverage but slower onset and offset kinetics. While optogenetic approaches require specialized light delivery systems, chemogenetic methods leverage simple drug administration without hardware but trade off fine temporal and spatial resolution. Their integration offers a compelling strategy for achieving both precision and flexibility in neuromodulation. As discussed by Vlasov^[^ ^225^ ^]^ et al. and Li et al.,^[^ ^226^ ^]^ the complementary nature of these technologies allows for their synergistic use, for example, using optogenetics to precisely stimulate specific neuronal populations while employing chemogenetics as a ligand‐controlled safety switch or to modulate broader circuit activity. The combined use of optogenetics and chemogenetics may enhance therapeutic outcomes by offering both circuit‐level specificity and systemic modulation. This integration opens up new possibilities for dissecting complex biological processes and developing advanced therapeutic interventions.

### 4.3 Clinical Applications

**Figure 4:**
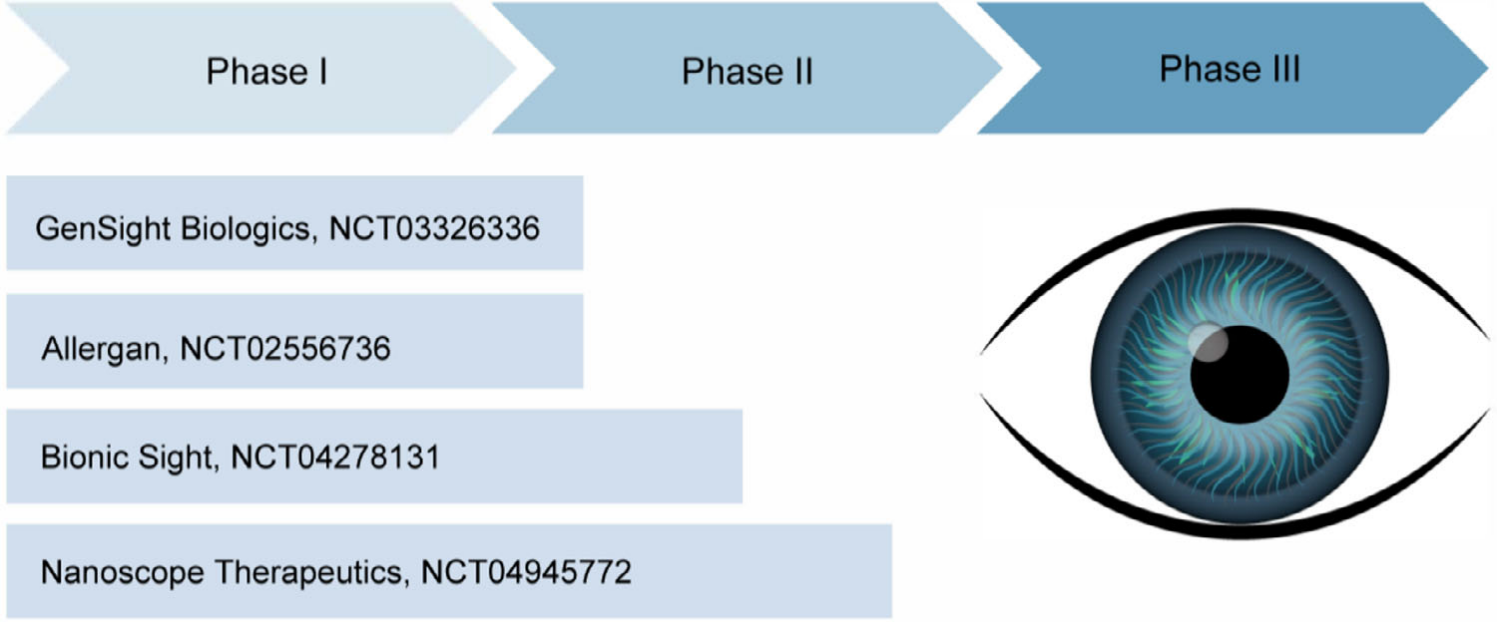
Clinical trials of optogenetic therapy for vision restoration. Ongoing intravitreal rAAV2‐mediated optogenetic trials for retinitis pigmentosa employ microbial opsins to restore light sensitivity: ChrimsonR (GenSight Biologics), ChR2 (Allergan), ChronosFP (Bionic Sight) and MCO1 (Nanoscope Therapeutics).

As discussed in Section 3.4, optogenetics offers therapeutic promise for diverse conditions including cardiac arrhythmias, psychiatric disorders, and cancer, while concrete clinical applications are already emerging, most notably in inherited retinal diseases. One of the most notable milestones in this regard was the first successful human trial in 2021, where a patient with retinitis pigmentosa regained partial vision through ChrimsonR‐based optogenetic therapy.^[^ ^40^ ^]^ This therapy involved (adeno‐associated viruses) AAV‐mediated gene delivery of the ChrimsonR channel to make the retina's remaining cells light‐sensitive, offering a promising approach for vision restoration. Currently, several ongoing clinical trials^[^ ^227^ ^]^ are investigating the use of optogenetic tools to treat Retinitis Pigmentosa (**Figure** **4**).

The eye proved to be the “lowhanging fruit” for optogenetic therapies for three main reasons: its optical accessibility behind the transparent cornea and lens allows noninvasive or minimally invasive light delivery; its subretinal space is relatively immuneprivileged and small, limiting inflammatory risk and vector doses; and its circuitry is simple, requiring only a single rhodopsin gene to restore light sensitivity in inner retinal neurons without rebuilding complex networks. Looking forward, the next accessible clinical frontiers include cochlear optogenetics for hearing restoration, where spiral ganglion neurons form a tonotopic array accessible via a small opening and channelrhodopsins can drive auditory nerve firing with millisecond precision; and cardiac pacing, in which implantable LEDs or catheter‐based fibers can deliver light to the sinoatrial node for cell type‐specific rhythm control. Emerging opportunities also span motor neuron diseases, where spinal or peripheral motor neurons could be selectively activated to restore muscle function; and vegetative state management via optogenetic modulation of autonomic or arousal circuits. By targeting organs and pathways with favorable light penetration, limited volume, and straightforward circuitry, optogenetic therapies can realistically advance into pilot human trials for hearing loss, cardiac arrhythmia, motor neuron disorders, and vegetative state intervention before addressing deeper or more complex systems such as the brain or systemic immune modulation.

## 5 Challenges and Considerations

While optogenetics holds remarkable potential, several challenges remain, especially concerning in vivo applications and clinical translation.

### 5.1 Engineering New Tools and Functional Limitations

It is important to recognize that optogenetics can control a limited range of cellular events due to the functional constraints of the available tools. To achieve comprehensive and convincing outcomes, optogenetic techniques often need to be combined with other methods. For example, optogenetics may be paired with chemical biology techniques, pharmacological interventions, or imaging methods like calcium imaging and voltage‐sensitive dyes. This combination allows for a more precise and holistic understanding of cellular dynamics. Integrating complementary approaches is crucial for addressing the limitations of optogenetic tools and enabling more nuanced control and observation of complex biological processes. On the other hand, the design and engineering of novel optogenetic tools with enhanced functionality remain vital but are inherently difficult and time‐consuming. These tools must be fine‐tuned for various properties, such as ion specificity, kinetics, and light sensitivity. While developing new tools is essential for expanding the application of optogenetics in diverse biological contexts, it presents significant technical challenges, especially when aiming for high precision in distinct cell types or organ systems. By acknowledging the inherent limitations of optogenetics, researchers can more effectively strategize how to combine it with other techniques, thereby producing more reliable and interpretable results in both basic and clinical research.

### 5.2 In Vivo Application Challenges

Optogenetics is increasingly being applied in vivo, and the use of advanced optical technologies such as red‐shifted rhodopsins and implantable µLED devices enables the control of cellular processes in living animal models with greater precision. Delivering and activating optogenetic actuators effectively within live organisms presents several technical hurdles. Light penetration in tissue is limited by scattering and absorption, with blue light attenuating within hundreds of microns.^[^ ^228^ ^]^ Achieving sufficient light penetration, particularly in deeper tissues or larger animal models, remains a key obstacle. While red‐shifted rhodopsins with enhanced light sensitivity improve tissue penetration, more advanced optical tools are necessary to deliver light with precision and minimize damage. Magnetogenetics bypasses the limited tissue penetration of light by leveraging magnetic fields that traverse biological tissues unimpeded. The “Magneto” system, comprising ferritin‐tagged TRPV4 channels, allows noninvasive neuronal activation with static or oscillating magnetic fields, though its kinetics are on the order of seconds and expression can be inconsistent.^[^ ^229^ ^]^ Despite these advances, magnetogenetic toolkits remain limited compared with optogenetics, with ongoing efforts needed to improve specificity, expression consistency, and temporal resolution.^[^ ^230^ ^]^ Equally important is the efficient and targeted delivery of optogenetic tools to specific tissues. Ensuring that optogenetic actuators are expressed with high specificity in the desired cells is a significant challenge, requiring advancements in gene delivery techniques such as AAVs and tissue‐specific promoters. In blind mouse models, targeted expression of ChR2 in rod bipolar cells successfully restored both ON and OFF light responses with high spatial acuity, demonstrating that optogenetic intervention at the level of secondary retinal neurons can reconstitute complex visual signaling pathways.^[^ ^231^ ^]^ Avoiding off‐target effects and achieving efficient expression are critical for successful in vivo applications. Overcoming these barriers will require multidisciplinary collaboration among bioengineers, neuroscientists, and geneticists to refine both optical and genetic delivery methods, ensuring more reliable and targeted in vivo optogenetic interventions.

### 5.3 Long‐Term Safety and Immunogenicity Concerns in Gene Therapy

As optogenetics advances toward gene therapy applications, ensuring long‐term safety and addressing immunogenicity concerns are critical. Many optogenetic proteins, like ChRs, originate from non‐human species, which increases the potential for immune responses when introduced into human patients. Potential immune reaction poses a significant risk for long‐term therapies, where the body might reject these foreign proteins over time or exhibit unforeseen toxic effects. Furthermore, the irreversible nature of genetically transfected neurons or implanted devices adds complexity, as there are limited options for removal if complications arise. Immunogenicity of microbial rhodopsins can provoke cellular immunity over time; mitigating tactics include humanizing rhodopsin sequences, transient immunosuppression regimens, and using tissue‐restricted promoters to limit off‐target expression.^[^ ^232^ ^]^ Long‐term safety concerns, such as phototoxicity, thermal damage, and chronic rhodopsin overexpression, are being addressed through low intensity, pulsed stimulation protocols, enhanced rhodopsin kinetics to minimize light exposure, and thorough histological monitoring in animal models.^[^ ^233^ ^]^

With ongoing advancements in precision tool development, in vivo techniques, and clinical applications, optogenetics is set to continue transforming our understanding of biological systems and opening up new possibilities for treating diseases. The road ahead requires careful consideration of these hurdles, but the potential of optogenetics remains as promising as ever.

## Conflict of Interest

The authors declare no conflict of interest.

## Author Contributions

X.D. and M.Z. contributed equally to this work. S.G. drafted the initial version of the manuscript. X.D. created the figures. All authors contributed to the revision process and approved the final version for submission.

## Peer Review

The peer review history for this article is available in the Supporting Information for this article.

## Supporting information

Supplementary Information: Record of Transparent Peer Review
