# Supplementary material for: Two Decades of Optogenetic Tools: A Retrospective and a Look Ahead
Source: Adv Genet (Hoboken). 2025 Sep 2;6(3):e00021. doi: 10.1002/ggn2.202500021 (PMC12482941; doi:10.1002/ggn2.202500021)
Supplement: Supplementary file 1 — Supplementary Information: Record of Transparent Peer Review [file GGN2-6-e00021-s001.docx]

Record of Transparent Peer Review

Two Decades of Optogenetic Tools: A Retrospective and a Look Ahead

Data submitted: 26-Apr-2025

1st Editorial Decision: 07-Jun-2025

1^st^ Revision Received: 20-Jul-2025

2^nd^ Editorial Decision: 30-Jul-2025

2^nd^ Revision Received: 04-Aug-2025

Accepted: 07-Aug-2025

Revision with responses: 20-Jul-2025

***Reviewer #1***

1. The subsections within some chapters (e.g., 2.1, 2.2) are overly detailed, with some content overlapping or repeating. Consider consolidating these sections to emphasize the main narrative. For example, descriptions of ChR2 variants appear multiple times in different paragraphs and could be combined and summarized.

Reply: Thank you for your valuable feedback. In response, we have carefully reviewed and consolidated the subsections within chapters such as 2.1 and 2.2. Descriptions of ChR2 variants and related content have been significantly shortened and merged to reduce redundancy and improve clarity, while still preserving key information relevant to the overall narrative.

2. Figures and tables (e.g., Figure 1, Figure 2, Table 2) are mentioned frequently, but the text provides limited interpretation and analysis of these visuals. Expand on the interpretation of key figures and tables in the main text to highlight their supportive role in the review's central argument.

Reply: We appreciate the reviewer’s suggestion and fully agree that figures and tables should be more effectively integrated into the main text. In the revised manuscript, we have expanded the interpretation and discussion of Figure 1, Figure 2, and Table 2 to better highlight their relevance to the review's central themes. Additionally, we have modified Figure 2 to make it more informative and visually coherent, and we have revised several details in Table 2 to improve clarity and completeness. These updates aim to strengthen the explanatory value of the visuals and enhance the overall readability of the manuscript.

3. While the review covers major optogenetic tools such as ChRs, HRs, PACs, ACRs, and KCRs, it lacks sufficient detail on emerging or less common tools (e.g., lightcontrolled protein degradation systems, light-controlled splicing, light-controlled RNA regulation). Consider adding content on these or mentioning them in the future perspectives section.

Reply: We appreciate the reviewer’s suggestion. In response, we have expanded the review to include additional emerging optogenetic strategies. In Section 2.4.2, we now discuss light-controlled protein degradation systems in more detail, including the ROSindependent degradation approach and a brief mention of photocaged molecule-based methods. In Section 2.4.3, we introduce optogenetics-enabled drug screening platforms as a growing application area. Furthermore, a new subsection 3.3 titled LightControlled Splicing and RNA Regulation has been added to highlight recent advances in optogenetic control of RNA metabolism, splicing, and translation. These additions aim to broaden the scope of the review and reflect the rapid diversification of optogenetic technologies beyond classical tools.

4. The review does not thoroughly discuss the combined applications of optogenetics with other regulatory technologies like CRISPR and chemogenetics. Expand on this aspect.

Reply: We appreciate the reviewer’s suggestion to highlight combined optogenetic–regulatory approaches and have accordingly expanded our coverage. In Section3.3, we now include examples of light controlled CRISPR systems. In Section4, we expanded the 4.2“Multiplexing Techniques” part and discussed hybrid strategies combining optogenetics with chemogenetics and with CRISPR tools . These additions underscore the versatility of multi modal approaches for dynamic, reversible, and highly specific control over cellular function, while also discussing current technical challenges and future opportunities.

5. Although the review mentions cardiovascular, hematological, and botanical fields, the examples and depth are significantly less than in neuroscience. Increase the application examples and future prospects analysis in non-neural systems (e.g., immunology, metabolism, development, oncology).

Reply: We thank the reviewer for this important comment. In response, we have expanded the discussion of non-neural applications of optogenetics in Sections2.3, 2.4, 3.3, and 4, including new examples from immunology, metabolism, development, and oncology. However, we must also acknowledge that optogenetics was originally developed and has matured primarily within the neuroscience field. While applications in other systems are growing rapidly and showing great promise, the breadth and depth of work outside the nervous system are still comparatively limited. We have revised the manuscript to better reflect both the emerging progress and current limitations in non-neural applications.

6. The clinical translation section primarily focuses on retinal diseases. Broaden the discussion to include potential clinical applications of optogenetics in other diseases (e.g., cardiac arrhythmia, Parkinson's disease, psychiatric disorders).

Reply: Thank you for this important suggestion. Although inherited retinal disorders are currently the only indication in which optogenetics has entered clinical trials, and thus formed the focus of our discussion, we have expanded Section3.4 to highlight emerging applications in cardiac arrhythmias, neuropsychiatric and Parkinson’s disease models, hearing restoration, as well as immuno oncology. In Part4.3, we now also outline a strategic roadmap for advancing these modalities toward future human trials.

7. The limitations of optogenetic tools (e.g., light penetration depth, tissue-specific expression, immunogenicity, long-term safety, gene delivery efficiency) are mentioned but analyzed superficially. Provide a more in-depth analysis of the mechanisms underlying these challenges and current strategies to address them, incorporating the latest research.

Reply: We appreciate the reviewer’s call for deeper analysis of optogenetic limitations. Although detailed, in depth datasets on some of these topics are not yet available in the literature, in the revised manuscript, we have expanded the discussion to more detail, and have added related references.

8. The analysis comparing the advantages and disadvantages of optogenetics with other regulatory technologies (e.g., chemogenetics, drug-inducible systems) is limited. Include more comparative information.

Reply: Thank you for this suggestion. We have now expanded Section 4.2 “Multiplexing Techniques,” to include a detailed comparison of optogenetics with chemogenetics.

9. The references are mainly concentrated on recent mainstream journals. Supplement the references with citations of earlier foundational works to provide a more complete historical context of the field.

Reply: We agree on the importance of grounding our review in the foundational literature. Accordingly, we have added several more references. Given the vast and rapidly growing body of optogenetics research, we have to say that it is challenging to include every seminal work without diluting focus.

10. The introduction of ChR2 and its variants appears repeatedly across different sections. Streamline and merge these descriptions to eliminate redundancy.

Reply: Thank you for pointing this out. We have streamlined all instances of ChR2 and its variant descriptions, merging overlapping sections and reducing redundancy to improve clarity and flow.

11. Standardize terminology throughout the review (e.g., "opsin," "rhodopsin," "lightsensitive protein") and define terms upon their first use.

Reply: We appreciate the reviewer’s emphasis on consistent terminology. Throughout the manuscript, we have standardized key terms.

***Reviewer #2:***

"Review Summary Title: Two decades of Optogenetic tools: A retrospective and a look ahead Recommendation: Major Revision Strengths: Comprehensive coverage of optogenetic tools across ion channels, signaling molecules, and protein interactions. Forward-looking discussion of clinical applications, subcellular targeting, and red-shifted tools. Includes recent developments like KCRs, OptoGPCRs, and all-optical methods.

Reply: We thank the reviewer for highlighting the comprehensive parts in our manuscript, as well as the forward-looking discussion. We are also grateful for the acknowledgment. These positive evaluations encourage us to further refine and strengthen the review.

Weaknesses Overly descriptive: Lacks critical comparison of tools' performance (e.g., kinetics, ion selectivity, tissue penetration).

Reply: We appreciate the reviewer’s point regarding the comparative analysis of tool performance. Our primary goal was to provide a broad, integrative overview of the full spectrum of optogenetic actuators within the space constraints of this review. Detailed comparisons of kinetics, ion selectivity, and tissue penetration have been the focus of several dedicated reviews. In response to this comment, we have added signposts in the text directing readers to these specialized sources for in depth analysis of these parameters, at the end of part 2.1 and 2.3.

Redundancy: Repetitive mentions of ChR2 variants and clinical use cases dilute clarity.

Reply: We thank the reviewer for identifying redundancy in our discussion of ChR2 variants and clinical examples. We have carefully consolidated repeated descriptions, merged overlapping sections, and refocused the narrative to improve clarity and flow. The clinical use part is also modified according to the advice of both reviewers.

Poor figure design: Figures are underexplained and hard to interpret.

Reply: We appreciate the reviewer’s feedback regarding figure clarity. In the revised manuscript, we have expanded the explanations in the main text and enhanced figure captions to guide interpretation. Additionally, some of the figures have been modified to ensure that they more clearly convey the key concepts and data.

Missed synthesis: No framework for tool selection or evaluation; minimal analysis of trade-offs.

Reply: We thank the reviewer for this insight. Our primary goal was to offer a broad, integrative overview rather than prescribe a one size fits all framework for tool selection, since optimal choice also depends heavily on specific experimental parameters. To aid readers, we linked to external resources (e.g., OptoBase) and specialized reviews where more detailed decision making frameworks are provided.

Dense language: Text is overly technical in parts and lacks transitions or visual aids.

Reply: We appreciate the reviewer’s observation regarding the manuscript’s language density. In the revised version, we have simplified overly technical passages and added clearer transitions where we think necessary. These changes are intended to make the content more accessible without compromising scientific accuracy.

Suggestions: Add benchmark table comparing major tool classes (e.g., ChR2 vs. ReaChR vs. KCR1).

Reply: We have included major tool classes in Table2. To avoid redundancy, as noted by Reviewer1 for the rhodopsin part, we have not expanded much into subtype variant. Instead, we direct readers to specialized resources (e.g., OptoBase) or reviews for detailed profiles of individual subgroups.

Clarify figures and reduce redundancy.

Reply: We’ve updated the figure explanations and removed redundant elements.

Provide critical insights, not just historical summaries.

Reply: Thank you for this suggestion. We have enhanced Section5 to incorporate critical insights.

Discuss limitations and alternatives (e.g., chemogenetics, magnetogenetics)."

Reply: We have now expanded the manuscript in Part4 and 5.2 that more fully discusses the limitations of optogenetics and alternative modalities, such as chemogenetics and magnetogenetics, highlighting their respective advantages, trade offs, and opportunities for hybrid approaches.
